# Characterization and Identification of Natural Terpenic Resins employed in “*Madonna con Bambino e Angeli*” by *Antonello da Messina* using Gas Chromatography–Mass Spectrometry

**DOI:** 10.1186/1752-153X-6-59

**Published:** 2012-06-21

**Authors:** Mario Vincenzo Russo, Pasquale Avino

**Affiliations:** 1 Facoltà di Agraria (DISTAAM), Università del Molise, via de Sanctis, Campobasso, 86100, Italy; 2 DIPIA, INAIL ex-ISPESL, via Urbana 167, Rome, I-00184, Italy

**Keywords:** Binding media, Paintings, Natural resins, Marker, GC-MS, Madonna with the Infant and Angels, Antonello da Messina.

## Abstract

**Background:**

Natural resins were frequently employed as adhesives or as components of oleo-resinous media in paintings in the past. The identification of vegetable resins is still an open problem. The aim of this paper is to analyze by GC-MS some vegetable resins frequently employed in paintings, such as Venice turpentine, dammar, copal, elemi in order to identify their main component in raw and aged samples. Some molecules are proposed as chemical “*markers*” to identify these natural resins.

**Results:**

The results obtained on standards allowed us to successfully analyze sample collected from one work of art: the *Madonna with the Infant and Angels* by Antonello da Messina (XV century).

**Conclusion:**

The results obtained confirm that the painting the artist originally used a mixture of linseed oil and natural resin (Venice turpentine) as binding medium.

## Background

The identification of the compounds employed in artistic paintings provides valuable information for both the knowledge of an artist and a correct restoration project. In particular, it is the analysis of binding media that allows it; simultaneously, this issue has always been one of the most important goals of analytical chemistry in conservation studies [1-4]. Identification of binding media involves several analytical problems mainly related to the very low amount of sample available and its heterogeneity, and very low content of organic media components as well as unknown state of alteration/degradation.

**Table 1:** Characteristic fragment ions and corresponding m/z values of dehydroabietic acid (DHA) and 7-oxo-dehydroabietic acid (7-oxo-DHA) as methyl esters

**Ion**	**DHA**	**7-oxo-DHA**
M^+·^	314	328
[M - CH_3_]^+^	299	313
[M - COOCH_3_]^+^	255	269
[M - HCOOCH_3_]^+^		268
[M - CH_3_HCOOCH_3_]^+^	239	253

A difficult but challenging task in these studies is the identification of markers. These are characteristic compounds that allow the precise identification of a resin or of a drying oil in a sample taken from a painting. Indeed, to reveal the presence of a particular substance used by an artist, it is necessary to find out suitable markers.

Binding media are natural products of relatively complex nature that are used as film-forming substances to give cohesion to pigments, to adhere them to each other and to a backing substrate and to protect them from deterioration [5-10]. Analytical chemistry offers suitable techniques to provide the restorer with the necessary analytical information for conservation treatment [11].

Binding media are suitable for a classification based on their chemical nature. Linseed oil is the most widely employed drying oil. Among proteinaceous substances, the most commonly used are: egg yolk, egg white and casein. Glues of animal origin have often be seen employed, both by artists and restorers: they are mainly constituted from collagen and keratin. Among the proteinaceous glues, beef and porcine gelatines, albumin, casein and egg protein are the most widely employed as binding media in tempera paintings [12-16].

Natural resins of vegetal origin were widely employed in easel painting [17]. Different natural resins were mainly used for decorative and varnish purposes since very ancient times for their excellent glasslike capability. A great number of recipes and preparation procedures are documented in literature [18,19]. Artists used them as final varnish for their protective properties and with the aim of improving optical properties of painting films. Sometimes resins were added to drying oils to modify the fluidity of the binding media. Resins form a protective coat over a paint film and give an uniform surface to the work. Besides, they improve the optical qualities of the picture by increasing both the saturation of the color and the overall gloss.

Among the various class of organic materials used as binding media, the natural resins are those which still have the reputation of being difficult to identify and suffer from analytical neglect [12,16]. Natural resins are secreted by a large variety of plants. They are largely composed by mono-, sesqui-, di- and triterpenoids. Interesting and very useful for classification and identification, is the fact that di-and triterpenoids are not found together in the same resin [15]. Both artists and restorers have largely used, in virtually all parts of paintings, diterpenic resins. Venice turpentine and Strasbourg turpentine were most popular. Pine resins, first of all colophony, presented the problem of becoming dark and brittle. Nevertheless sources suggest that pine colophony-drying oil mixtures where frequently used [20]. Triterpenoids can be either tetracyclic or pentacyclic. The tetracyclic compounds are based either on the dammarane skeleton or the euphane skeleton. The most popular triterpenic resins used in painting are dammar and mastic [19,21-23].

This paper focuses on the application of chromatographic techniques to the preservation and conservation of one of the most important paintings of the world cultural heritage. Gas chromatography–mass spectrometry (GC-MS) was employed, as it is a well-established technique for the analysis of complex mixtures and holds a prime position in analytical chemistry because of its combination of sensitivity, wide range of applicability and versatility [24]. In particular, a painting of the XV century, the *Madonna with the Infant and Angels* (“Madonna con Bambino e Angeli”) by Antonello da Messina, was studied and the composition of the natural resins of the binding media was investigated. From the artistic point of view, Antonello da Messina was the first Italian artist to use the oil painting in Italy due his strong relationship with Flemish masters.

In particular, drying oils and natural resins of vegetal origin were studied in order to get information about ageing processes that consist in modification and degradation of these compounds. The study was focused on drying oils and varnishes. The aim was to determine the drying oils and the resins originally employed by the artist and by restorers. Micro Fourier Transform-Infrared spectroscopy (FT-IR) analyses were also performed as a preliminary screening test to detect the presence of oleo-resinous material, before GC-MS analysis.

## Results and Discussion

**Figure 1:**
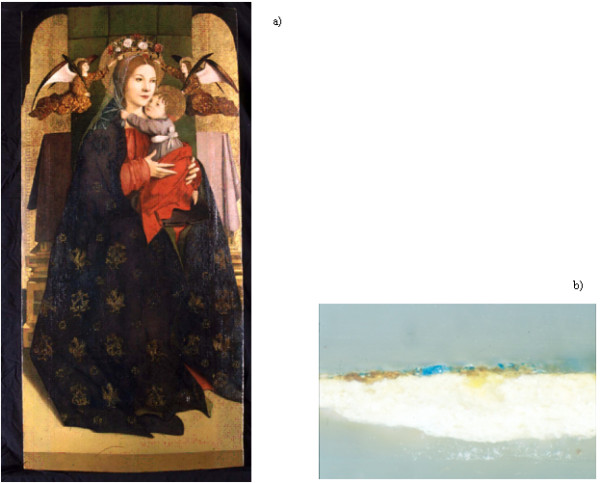
**a: “*Madonna con Bambino e Angeli*” by Antonello da Messina (XV century; Uffizi Gallery, Florence). b**: layer from which the sample was taken.

Analytical procedures, developed and tested on standards, were applied to analyze samples collected from old masters paintings that where in restoration by the Istituto Centrale per il Restauro (ICR) of Rome, Italy. In particular, the “*Madonna con Bambino e Angeli*” by Antonello da Messina (XV century) was considered (Figure1a).

Firstly, a micro-FT-IR was used because it proves to be a powerful tool to obtain significant and non-destructive analyses on different materials, both organic (such as binders, fibers, polymers) and inorganic (pigments and fillers for instance) [25-28]. This technique was employed as a preliminary analytical technique to detect the presence of resinous materials in a sample collected from the painting of artistic interest “*Madonna con Bambino e Angeli*”, by Antonello da Messina.

FT-IR spectra of the most commonly used, in fine arts, drying oils (e.g., linseed oil) and terpenic resins (e.g., Venice turpentine, Manila copal, dannar, mastic) were collected, in order to identify diagnostic bands.

Spectroscopic analyses were performed on standards of fresh and aged resins. FT-IR spectra of natural terpenic resins are characterized by typical absorption bands, according to their molecular functional groups. Typical spectra of natural resins show a broad band in the 3500 cm^-1^ region due to the stretching of OH groups, absorption of C = O and C-O groups occur respectively at 1715–1695 cm^-1^ (strong) and 1240 cm^-1^ (weak). Methylic and methylenic groups give two sharp, strong absorption in the ranges 2960–2930 cm^-1^ and 2875–2865 cm^-1^ (C-H stretching) while 1467–1448 cm^-1^ and 1387–1382 cm^-1^ bands are due to the C-H bending [29].

The aged linseed oil, indeed, shows the following characterizing bands: 3400, 2930, 2855, 1780, 1735, 1713, 1459, 1418 cm^-1^ (strong), 1245, 1178 cm^-1^ (medium) and 1097, 980, 725 cm^-1^ (weak).

The analysis of a sample extracted by solvent action on a sheet of Japanese paper, according to the usual restorers’ practice [30,31], is an interesting example of the micro FT-IR potentialities. The analyses were carried out directly on a single fiber of the paper crashed on the diamond cell. The IR beam was focused first on a sample spot, then on a clean fiber spot. The FT-IR spectrum of the varnish was obtained subtracting the spectrum of the blank fiber from the spectrum containing both the varnish and the cellulose absorption bands.

The obtained spectrum is characterized by the following absorption bands: 3400 cm^-1^ (broad) due to the OH stretching; C = O stretching occurs at 1780, and 1730 (as shoulders) due to the presence of drying oils and 1707 cm^-1^ (strong) can be ascribed to resins. In the finger-print region the following bands occur: 1460 and 1384 cm^-1^ (medium), 1318, 1186, 1135 cm^-1^ (weak).

**Figure 2:**
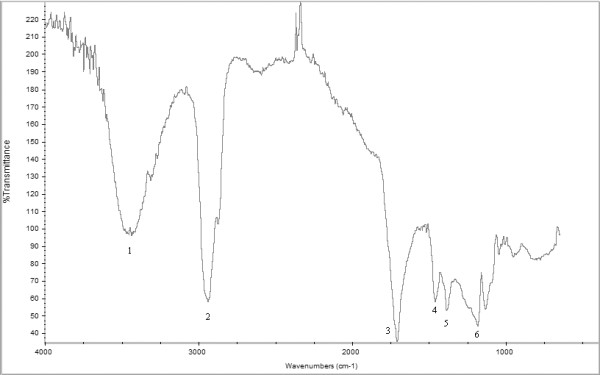
**Micro-FTIR spectrum of the varnish from the painting, obtained by spectral subtraction of the fiber of Japanese paper from the sample.** Signals: 1: OH stretching; 2: C-H bending of methylic and/or methylenic groups; 3 and 6: C = O and C-O stretching; 4 and 5: C-H bending of methylic and/or methylenic groups.

In the case of the painting by Antonello da Messina, it was possible, analyzing a single fiber from the Japanese paper employed to extract by solvent action the varnish, to get meaningful information on the oleo-resinous varnish extracted from the painting and on the cellulose of the fiber at the same time (Figure2).

It is however possible to identify the chemical class of organic materials by the diagnostic bands. Here the presence of drying oils and terpenic resins was verified.

Micro-FT-IR analyses did not permit, however, to identify directly the kind of resin or of drying oil employed in a paintintheg, but allows us to address the subsequent analyses in order to optimize the employment of the micro-samples, avoiding losses of scarce and precious materials.

**Figure 3:**
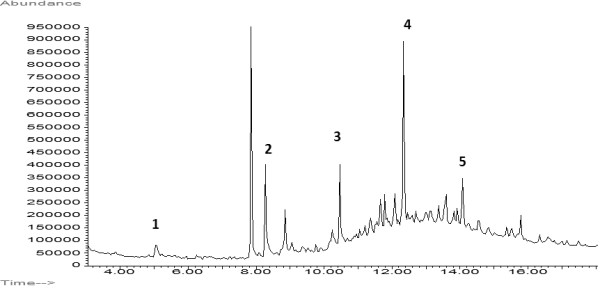
**TIC “*Madonna con Bambino e Angeli*” by Antonello da Messina.** 1: azelaic acid; 2: palmitic acid; 3: stearic acid; 4: dehydroabietic acid; 5: 7-oxo-dehydroabietic acid. For experimental condition: see the text.

Figure3 shows the Total Ion Chromatogram (TIC) of a sample taken from the painting by Antonello da Messina. The sample was collected from a layer (Additional file 1: Figure S1) corresponding to the binding medium (Figure1b).

The study and the characterization of binding media represent important aspects for painting characterization. In oil painting the binding medium used by artists is a drying oil which allows effective color application and helps in providing optic properties to pigments in drying place. Linseed oil, walnut oil and poppyseed oil are traditionally used in paintings. Different characteristic can be considered for identifying a binding medium. Therefore, one or more characteristic components of the oil used, should be detected. For example, a lipidic fraction may be characterized according to certain mono- or di-carboxylic fatty acids, or by using the ratio between azelaic acid and palmitic acid, or the one between palmitic acid and stearic acid. During ageing, drying oils show the production and progressive increase of dicarboxylic acids, while saturated fatty acids are so stable that they are not involved in the oil polymerization process. The palmitic and stearic acid concentrations change due to the evaporation over time but their ratio remains more or less stable. Several authors have evaluated the efficiencies of the ratio between palmitic acid and stearic acid (P/S) as an identifying parameter of linseed, walnut and poppyseed oil. They found P/S values included in the range 1.4-1.9 for linseed oil [20,32-34]; 2.4-2.9 for walnut oil [20,32-34]; 2.9-3.7 for poppyseed oil [34-38]. Starting from these considerations, over the chromatographic profile above reported, we found presence of both saturated acids (palmitic acid and stearic acid) and the decomposition products of the oil painting film (azelaic acid). In particular, the P/S ratio (1.3) suggests the employment of linseed oil in this painting.

Actually, also palmitic acid/azelaic acid (P/A) and palmitic acid/oleic acid (P/O) ratios are important *markers* of the ageing process but it will be not discussed in this paper.

**Figure 4:**
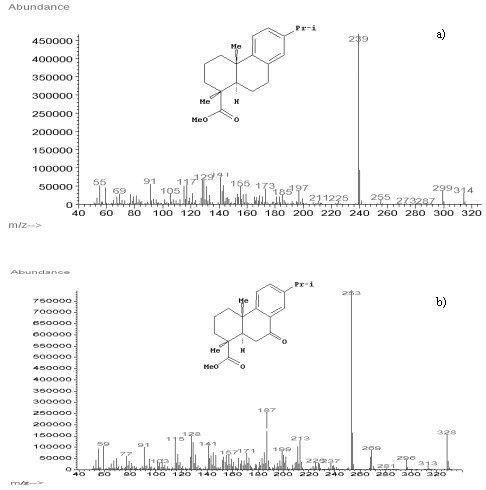
Mass spectra and molecular structures of dehydroabietic acid methyl ester (a) and 7-oxo-dehydroabietic acid methyl ester (b).

**Figure 5:**
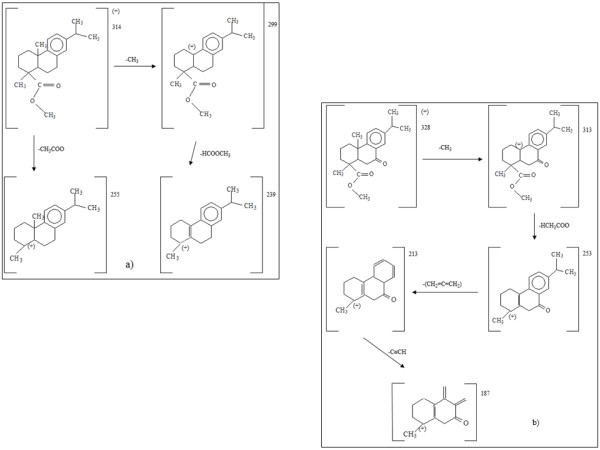
Fragmentation pattern of dehydroabietic acid (a) and 7-oxo-hydroxydehydroabietic acid (b).

A similar characterization was performed on the natural resin added to oil paintings for increasing flexibility in oil binders. A previous study [39] reported the detection of some characteristic molecules considered *markers* for natural resins. In particular, among the diterpenic resins, dehydroabietic acid and 7-oxo-dehydroabietic acid are characteristic for Venice turpentine. The mass spectra along with the molecular structures of these compounds are reported in Figure4. The mass spectra of methyl esters of diterpenic abietic acids are characterized by a typical fragmentation pattern, which involves: a) the loss of the ester group; b) the expulsion of a methyl group (often in the C20 position); c) expulsion of water or methanol from the hydroxyl moieties. In Table 2 characteristic fragment ions are listed for dehydroabietic acid methyl ester and 7-oxo-dehydroabietic acid methyl ester. In Figure5 the fragmentation patterns for these two compounds are illustrated in more details.

**Figure 6:**
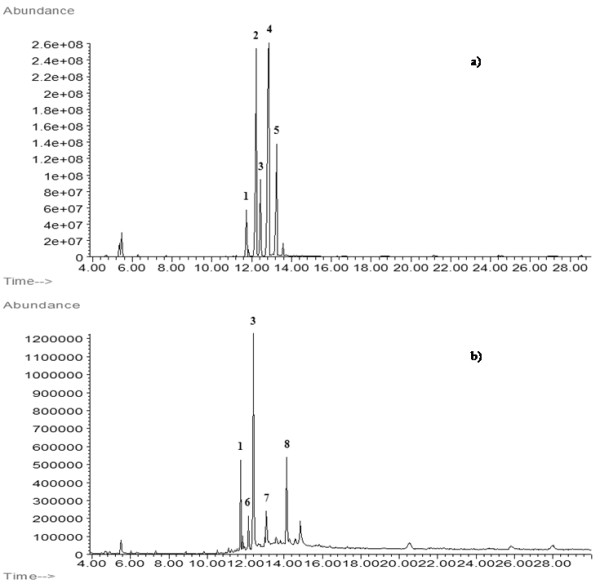
**Partial TIC of fresh (a) and aged (b) Venice turpentine.** For experimental condition: see the text. Peak number: 1: sandaracopimaric acid (Molecular Weight, MW, 316); 2: palustric acid (MW 316); 3: dehydroabietic acid (MW 314); 4: abietic acid (MW 314); 5: neoabietic acid (MW 316); 6: isopimaric acid (MW 316); 7: 6-dehydroabietic acid (MW 309); 8: 7-oxodehydroabietic acid (MW 328).

Figure6 show the TICs of raw (a) and aged (b) Venice turpentine, respectively: several compounds were identified in order to characterize this resin. Among them it was important to point out the species that survive to ageing processes, in order to find out suitable markers that allow us to recognize Venice turpentine in a sample. Thus, we analyzed artificially aged Venice turpentine. The artificial ageing process was performed by exposing specimens on glass of Venice turpentine, previously dissolved in methylene chloride, to the action of UV radiation for 22 days and then leaving the resin to ultimate the ageing process for 18 years in the dark [40-43].

It is interesting to notice that the composition of Venice turpentine changes significantly with ageing. The most abundant compound found in fresh resin is abietic acid because Venice turpentine is a valuable material used only in fine arts. Therefore larch resin, from which Venice turpentine is obtained, undergoes soft refining thermal treatments. Venice turpentine turns out to be softer and more malleable than other resins and retains the intense characteristic odor of the original resin.

Still, in aged resin we do not find trace of abietic acid. This is another proof of the tendency to disappear of this compound with ageing. In fresh Venice turpentine dehydroabietic acid is present in small amounts, while 7-oxo-dehydroabietic acid is absent. With ageing the situation changes and dehydroabietic acid becomes the main constituent, followed by 7-oxo-dehydroabietic acid. Another significant compound which survived ageing, turned out to be sandaracopimaric acid. By the comparison of the chromatogram of fresh and aged resin it is possible to identify dehydroabietic acid, 7-oxo-dehydroabietic acid as markers of Venice turpentine.

Finally, some authors report on characteristic compounds of Venice turpentine, as epimanool, larixol and larixyl acetate [15,20]. Actually, we did not find traces of these compounds neither in fresh samples of Venice turpentine, nor in aged ones. So we could not use these compounds as ulterior markers of the Venice turpentine.

In the painting investigated in this work, the TIC chromatogram reported in Figure3, shows the presence of the markers of the diterpenic resin of the *pinacea* family, Venice turpentine: methyl dehydroabietate and methyl 7-oxodehydroabietate. It is very interesting to notice that the sample was taken from an original painting layer since, when the sample was taken, the superficial varnish layer had already been removed.

So we could state that the artist originally used a mixture of linseed oil and Venice turpentine (natural resin) as binding medium.

## Experimental

### Materials

The reagent used were: dichloro methane, methyl alcohol (RS for HPLC), diethyl ether, KOH and HCl (Carlo Erba, Milano, Italy). Dammar resin and elemi (G-9877 and G-0263, respectively) were supplied by Sigma (Milan, Italy). Venice turpentine (art. 3230) was purchased from Zecchi (Florence, Italy). Both sandarac resin and Manila Copal were supplied by Morrone (Rome, Italy). A sample of resin exudates was collected from Larix deciduas as fresh raw material. Venice turpentine, dammar, elemi and Manila Copal were also used for specimens on glass. Resins spread on glass supports were artificially aged by the action of UV radiation. A 150 W quartz sheathed mercury vapor lamp “H” (Helios Italquartz s.r.l., Milano, Italy), maximum emission at 320 nm and 370 nm, was employed to irradiate natural resins for 22 days at the distance of 50 cm in a weathering case type UY 600 (Angelantoni Climatic Systems, Massa Martana, Perugia, Italy) at 25°C and 80% Relative Humidity (RH); then the above mentioned resins were allowed to ultimate their ageing process for 18 years in the dark.

A sample of the paint layer from an old master oil paintings on canvas, was taken. It was collected from the painting “Madonna con Bambino e Angeli” by Antonello da Messina (ca. 1470), housed in “Uffizi” Firenze (Italy), and analyzed (Figure1a). The sample was collected from the uppermost layer after that the superficial varnish layer has already been removed. This means that the sample was taken from an original painting layer (Figure1b).

### Sample preparation for GC-MS analyses of standard resins

The procedure followed for standard resins samples preparation involved the sample dissolution in dichloro methane and subsequent derivatization by diazomethane [44].

1 mL of dichloro methane was added to a known quantity (1 mg) of sample. After the complete dissolution of the sample, diazomethane in diethyl ether solution was added till the solution turned deep yellow for the excess of diazomethane. The solution was then evaporated to 150 μL to eliminate the diazomethane in excess under a mild flow of N_2_.

### Sample preparation for GC-MS analyses of material collected from paintings

The samples collected from the old masters paintings could contain both oils and resins, so a double step derivatization method was followed. This procedure involved the alkaline hydrolysis of the triglycerides contained in drying oils and subsequent derivatization of the fatty acids obtained, these were then turned into methyl esters by trans-esterification reaction [35] and extracted with dichloro methane. The methyl esters were extracted using n-hexane (0.5 mL × 3 times). The second derivatization step was performed by the addition of diazomethane in diethyl ether solution to the collected extracts to improve the derivatization for carboxylic functions of resin components.

100 μL of dichloro methane and 100 μL of KOH 2 M methanolic solution were added to the sample (< 0.1 mg). The solution was stirred for about 2 minutes and left to rest for 15 minutes at 60°C. Then 500 μL of a solution of HCl_conc_ and CH_3_OH (1:1) were added. The esters were extracted three times using 100 μL of dichloro methane. Few drops of diazomethane in ethyl ether solution was added to the collected extracts, according to the previously described procedure. The solution was evaporated to 150 μL under a mild flow of N_2_, and were analyzed by GC-MS.

### GC-MS apparatus

GC-MS analyses were performed on a HP-5890 Series II gas chromatograph coupled to a HP-5972 mass selective detector. A fused-silica capillary column with chemically bonded phase (SE-54, 5% phenyl-95%dimethylpolysiloxane) was prepared in our laboratory [45,46] with the following characteristics: 30 m × 250 μm i.d., N (theoretical plate number) 115,000 for *n*-dodecane at 90°C; K' (capacity factor) 6.7; d_f_ (film thickness) 0.24 μm; *u*_opt_ (optimum linear velocity of carrier gas hydrogen) 39.0 cm s^-1^, and UTE% (utilization of theoretical efficiency) 91%. The chromatographic conditions for GC-MS analysis were: injector temperature 300°C; transfer line temperature 280°C; initial oven temperature 120°C; isothermal for 1 min; 30°C min^-1^ up to 200°C and isothermal for 2 min; then 5°C min^-1^ up to the final temperature of 240°C. The carrier gas was helium at a flow-rate of 1.3 mL min^-1^. The GC-MS analysis was performed in the SCAN mode (mass range 50–550 amu at 70 eV). The terpenic compound identification was obtained by the comparison of mass spectra included in the library NBS 75 K.1 of the mass selective detector UP-5972. Spectra not included in above mentioned library were compared with mass spectra available in literature [20,47-52].

### Micro-FT-IR apparatus and analyses

Micro-FT-IR analyses were made on a Nicolet (Madison, Wisconsin ,USA) 510 P FT-IR spectrometer coupled to a Nicolet IR microscope, mod. NicPlan. A Mercury-Cadmium-Telluride (MCT) detector, cryogenically cooled, was used to examine the region from 4000–650 cm^-1^. Each recorded spectrum is the sum of sixty four scans collected at a resolution of 4 cm^-1^.

The samples, crushed in a sample compression cell with diamond windows (μSample PlanTM, mod. 0042–444, Spectra-Tech Inc., Stamford, Connecticut, USA), were analyzed in transmission mode. The diamond cell, containing the crushed sample, was mounted on the microscope stage at the focal point of the IR beam condenser for spectroscopic analysis.

## Conclusions

The study of molecular changes due to ageing processes is particularly important, because these transformations cause physical changes such as variation in solubility. This aspect of the problem has to be taken into account when treatments to remove varnishes are applied.

Micro FT-IR technique was sometimes employed as a preliminary screening test to detect the presence of organic materials, drying oils and resins in particular, in microsamples collected from the paintings. The information obtained through IR spectra allowed us to focus the chromatographic analysis on these kinds of substances, thus getting the maximum information on the painting, using the minimum amount of sample. This is a very important point as the first aspect to consider when we approach the study of a work of art is its preservation.

As natural resins could be found in binding media as trace components, it is important to test procedures to analyze resins efficiently. Moreover it could be useful to develop analytical methods to determine, at the same time, the presence of oils and resins in micro-samples drawn from paintings.

GC-MS analyses of the standards of oils, both fresh and artificially aged, allowed us to verify the importance of the ratio between palmitic acid and stearic acid as *marker* for the identification of the particular drying oil used.

The analytical procedure developed and the results of the study carried out on standard materials allowed us to identify the drying oil and, eventually, the varnish used in the painting of artistic interest (XV century).

All the considerations reported in this paper, should help the restorers, not only from the point of view of art history, but also from that of the chemical composition of the originally employed material in order to formulate a correct diagnosis of the state of conservation of a work of art.

## Competing interests

Both authors declare that they have no competing interests.

## Authors’ contributions

MV coordinated the study. PA and MV set up the analytical procedure using GC-MS. PA processed data. MV provided the comparison with other literature. PA edited the text and prepared the final draft of the paper. All the authors have read and approved the final manuscript.

## Authors’ information

^a^Full Professor, Faculty of Agriculture, Università del Molise, via De Sanctis, Campobasso, I-86100 Italy.

^b^Researcher, DIPIA, INAIL ex-ISPESL, via Urbana 167, Rome, I-00184 Italy.

## Supplementary Material

Additional file 1 **Figure S1.** Scheme of the cross-section of a typical Old Master painting, illustrating the composition of pictures layers. Click here for file
