# Supplementary material for: Characterization and Identification of Natural Terpenic Resins employed in “Madonna con Bambino e Angeli” by Antonello da Messina using Gas Chromatography–Mass Spectrometry
Source: Chem Cent J. 2012 Jun 21;6:59. doi: 10.1186/1752-153X-6-59 (PMC3464792; doi:10.1186/1752-153X-6-59)
Supplement: Additional file 1 — Figure S1. Scheme of the cross-section of a typical Old Master painting, illustrating the composition of pictures layers. [file 1752-153X-6-59-S1.doc]

**Supplementary Material**

**Characterization and Identification of Natural Terpenic Resins employed in “*Madonna con Bambino e Angeli*” by *Antonello da Messina* using Gas Chromatography-Mass Spectrometry**

**Mario Vincenzo Russo^a^ and Pasquale Avino^b^**

Corresponding author: Mario Vincenzo Russo, Facoltà di Agraria (DISTAAM), Università del Molise, via de Sanctis, 86100 Campobasso (Italy). Ph.: +39 0874 404631; Fax: +39 0874 404652; E-mail: [mvrusso@unimol.it](mailto:mvrusso@unimol.it)


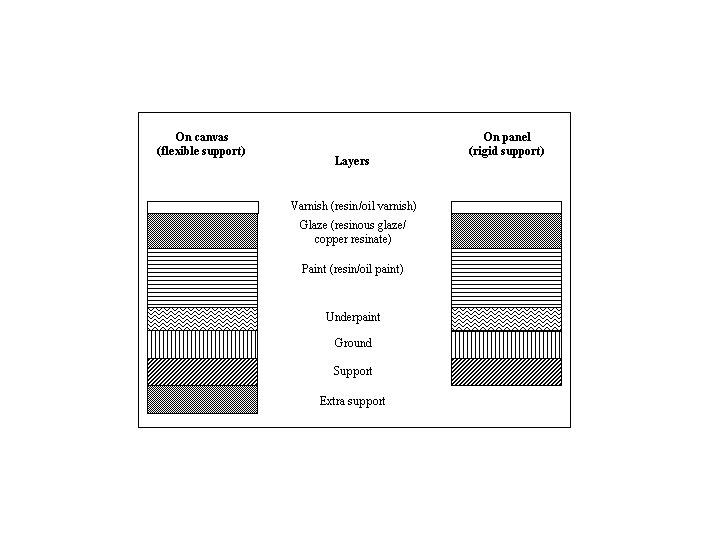


Figure 1. Scheme of the cross-section of a typical Old Master painting, illustrating the composition of pictures layers
